# Health sciences librarians’ awareness and incorporation of informed consent standards for medical image publication: a preliminary study

**DOI:** 10.5195/jmla.2026.1949

**Published:** 2026-10-01

**Authors:** Sarah McClung, Rachel Keiko Stark, Megan DeArmond, Sherli Koshy-Chenthittayil

**Affiliations:** 1 smcclung@ucdavis.edu, Head of Collection Strategies, University of California, Davis, Davis, CA; 2 stark@csus.edu, Health Sciences Librarian, Sacramento State University, Sacramento, CA; 3 mde_armo@touro.edu, Associate Professor and Systematic Review Services Librarian, Touro University Nevada, Henderson, NV; 4 skoshy-c@touro.edu, Data Analyst, Touro University Nevada, Henderson, NV

**Keywords:** Consent, images, publication, health sciences librarians

## Abstract

**Objective:**

The objective of this study was to determine if health sciences library workers were aware of informed consent best practices and standards for the publication of medical images and if they incorporated the recommendations into their professional work.

**Methods:**

A survey was developed by the authors to determine awareness and incorporation of informed consent best practices and standards for the publication of medical images. The survey was distributed via listservs of professional librarian associations in the United States. The results of the survey were tabulated in RStudio. Fisher's Exact Test was carried out to determine if awareness of informed consent influenced job responsibilities.

**Results:**

A total of 91 respondents were included in the data analysis with all respondents reporting multiple responsibilities (instructional, collections, and/or digital initiatives). While the majority of library workers (59%) were aware of the best practices and standards, few (6.6%) incorporated the recommendations into their professional work. Over 50% of librarians with instructional responsibilities, over 70% of librarians with collections responsibilities, and over 60% of librarians with digital initiative responsibilities were aware of the recommendations but did not incorporate the recommendations into their job duties.

**Conclusions:**

The professional work of health sciences library workers does not appear to include a significant incorporation of informed consent best practices and standards for the publication of medical images. There is a need for future research to better understand how library workers can better incorporate their knowledge of informed consent recommendations into their work.

## INTRODUCTION

### Health Professionals and Informed Consent

Photographs are commonly taken in medical settings to document clinical findings, especially in fields which specialize in conditions with primarily externally visible conditions, such as dermatology, plastic surgery, and maxillofacial surgery. Ensuring patient confidentiality, dignity, and autonomy are of paramount importance when disseminating these clinical images, including for the secondary or non-therapeutic purposes of education and publication [1–4]. The World Medical Association developed the Declaration of Helsinki, a global statement of ethical principles for medical research involving human subjects, to provide guidance on safeguarding identifiable patient data like clinical photographs [5]. De-identification of images is an essential component of ethical clinical photography and is heavily advised [6–10]. When a clinician wants to publish a patient’s photograph, but the patient's anonymity cannot be guaranteed via de-identification, obtaining informed consent is imperative to protect the patient from undue harm [1–4,6–9,11–18]. Informed consent is the ethical principle in medical treatment that ensures patients receive the necessary information to make informed decisions about their care and the use of their medical information [19].

The informed consent process must be transparent, documented in the medical record, and provide comprehensive information to patients regarding the purpose, risks, and potential outcomes associated with clinical photography. Obtaining explicit consent from patients for the use of their photographs is necessary to ensure they fully understand how their images may be utilized and disseminated. If consent is given for one specific purpose, it is not transferable to other uses and patients should have the freedom to decline ways their photo(s) could be disseminated, along with being photographed altogether [3,6,7,11,16–18,20]. In the United States, section 164.522 of the Health Insurance Portability and Accountability Act (HIPAA) gives patients the right to restrict the use of their protected health information, including identifiable medical photographs [21].

### Publishers and Informed Consent

Various professional associations have released recommendations and best practices regarding informed consent for the publication of patient photographs. Journal publishers most often cite [22,23] the International Committee of Medical Journal Editors’ (ICMJE’s) “Protection of Research Participants” [24] and the Committee on Publication Ethics’ (COPE’s) “Journals’ Best Practices for Ensuring Consent for Publishing Medical Case Reports” [25]. Even though these recommendations and best practices have existed for years and a few publications have updated their processes to improve patient privacy and autonomy [1,2,26], medical journal publishers have overall struggled to align their publication practices accordingly.

A 2022 scoping review examined the policies and practices of publishing identifiable photographs in scientific articles. The authors concluded that in 95% of the included studies, the consent process was not conducted properly by clinicians who intended to include medical photographs in research publications. The consent practices used were often considered insufficient or did not fully implement professional guidelines in practice. Healthcare professionals in the studies largely considered verbal consent to be sufficient and some even thought it was acceptable to publish patient photographs without consent [23]. Studies for specific disciplines included an analysis of top dentistry, oral surgery, and otorhinolaryngology journals which found that only 52% had a posted policy on the publication of clinical images [27]. Similarly, for dermatology, a review of the top ranked journals revealed that only 58% had explicit patient consent requirements [13]. While the feasibility of applying and enforcing publication guidelines and informed consent procedures, especially internationally, has been questioned [26], there have been many calls for implementation of clear protocols and standardization from clinicians, researchers, authors, and ethicists as a way to eliminate confusion, risk, and improve patient confidentiality [3,4,6,8,10–17,28–32].

### Technology and Informed Consent

The inconsistent application of informed consent guidelines and best practices is becoming increasingly problematic as technological advances are rapidly increasing the ease of taking, disseminating, and discovering clinical photographs. It is largely agreed that the rapid ubiquitousness of the smartphone was the catalyst for this significant disruption [1,3,8,11,15,18,30,33–36]. Historically, clinicians often relied on professionally trained medical photographers to take pictures of patients, but smartphones have enabled clinicians to take images whenever and wherever they like, largely bypassing the medical photographer [3,8,12,33,34,37,38]. There is evidence that the use of smartphones by clinicians has also led to a relaxation in informed consent practices. A shift towards obtaining patient consent verbally instead of in writing has been observed [3,8,33,37–39].

Before the widespread use of the internet, it was rare for a patient to discover their image(s) in the literature. Health sciences journals were primarily distributed as print publications to libraries and health professionals, making access and reproduction cumbersome. Now that almost all journals are available online and many are publishing at least hybrid open access (OA), a patient’s image can be copied and shared on the internet, including on social media, without the patient’s or publisher’s knowledge [1,14,18,27]. Clinicians and patients often do not review the platform’s privacy settings and terms of use when uploading photographs to social media and so they are not aware of how their images can legally be reused [18,40]. In one study, even though 70% of top dermatology journals had an account on one or more major social media platforms, only 6% required the disclosure of potential social media use of images in their publication consent process [22]. This is just one way social media has created ethical and legal complications regarding patient consent.

There is evidence that patients do not always understand that OA journal articles are just as widely available as other content on the internet. Publishing images in an OA article enables access to the photographs via the public domain, similar to sharing via social media, and often lacks the proper corresponding consent [4,23]. Not only is the image viewable, but it could be reused in ways which were never intended when a Creative Commons license, especially CC BY, is applied [1,23,27]. One very popular and potentially damaging way images in OA articles can be discovered is through the image database, Google Images. Marshall et al. concluded that if an article with a medical image is freely available online, the image is nearly three times more likely to be found on Google Images than an image behind a paywall [30].

The rapid developments in artificial intelligence (AI) have raised further concerns among researchers. Now that numerous computer programs can identify a person with high levels of accuracy without full-face images, clinicians should anticipate these advancements complicating patient privacy, informed consent, and previous definitions of identifiable [15,23]. Because of these complexities, it is recommended that, for patient images that are to be used for AI development, consent should explicitly include permission for this use [36].

Despite the overall benefits of OA, machine learning, and similar technological advances, these developments have compromised and will continue to compromise patients’ privacy and autonomy in ways never conceived [6]. One such example is the fact that a clinician’s inability to permanently delete a patient’s image from the public domain goes against a fundamental part of informed consent: the right for the patient to revoke consent at any time. Essentially, a healthcare provider is asking the patient to surrender their right to revoke consent when they disseminate their photograph via the internet [40]. As medicine and technology both evolve, the ethical issues related to patient privacy and confidentiality will not improve without consistent iteration of established codes of practice. The risks associated with new technologies need to be included in the informed consent process so that patients truly understand to what they are consenting [1,3,6,8,11,15,18,28,30,33–36].

### Effects of Unethical Dissemination of Medical Images

There is a risk, due to the unequal patient-provider power dynamic, that patients can feel as though they must agree to photography for treatment or to get the best care [4,11,12,16,32–34]. This dynamic can be intensified in environments where there are low resources, language and/or cultural differences, and vulnerable patient populations [11,32]. When identifiable photographs are shared for secondary purposes without the appropriate consent, it has the potential to cause not only legal trouble for clinicians, but psychological harm to the patient [6,8,16,34]. This is especially true for patients who belong to marginalized groups, such as the Lesbian, Gay, Bisexual, and Transgender Plus (LGTBQ+) and Black, Indigenous, and People of Color (BIPOC) communities, as these betrayals further erode patients’ tenuous trust in healthcare due to a long history of medical professionals taking advantage of such individuals.

Studies have been conducted which examine the effects on intersex and transgender patients, respectively, when their images are shared without their consent [16,30]. These two populations have historically experienced heightened rates of harassment, discrimination, and/or physical harm. The chance of incident would increase further if their identities were revealed to or pieced together by nefarious individuals via medical publications. The likelihood of this occurring is increased by the fact that there are a limited number of clinicians who provide healthcare to these patients. This limitation means that intersex and trans patients could be more easily identified using the author information in combination with photographs and case report descriptions [30]. The same can be true for patients with rare diseases [1].

A patient’s identity and history must be taken into consideration when seeking informed consent. A scoping review of patient perceptions of dermatological photography discovered consistent disparities among ethnic groups. Non-white patients expressed greater discomfort with medical photography and were more likely to believe medical photography would not improve their care. These differences were attributed to the non-white patients experiencing the negative effects of the systemic inequities and implicit biases prevalent in healthcare [29]. The heightened interest in properly obtained patient consent is part of the increasing worldwide demand for human rights, equity, and empowerment [32]. Providing and enforcing consent standardization could be a step towards improving consistency and equitability in patient care in relation to photography, privacy, and autonomy [6].

### Library Workers and Informed Consent

Another, often overlooked, player in the dissemination of medical photographs is health sciences library professionals. Librarians purchase and subscribe to resources for their users, including materials that may not abide by the established best practices and standards for informed consent. They then teach and encourage patrons to use these same resources. With or without their knowledge, health sciences library workers are perpetuating the use of unconsented images and, consequently, the harm inflicted on patients who did not provide consent for their images to be used. As of the writing of this publication, the authors could not find any peer-reviewed, published literature that addressed librarians’ awareness of informed consent best practices and standards for the publication of medical images and how they incorporate the recommendations into their professional work. This surprised the authors given the abundance of research and publications on the topic of informed consent and the publication of medical images.

The authors hypothesized that this lack of publication signaled that library workers might not be familiar with these standards and best practices. They decided to investigate these topics with health sciences library workers, as the most likely group within the library profession to have been exposed to these concepts in their professional work. The research questions developed by the authors were: 1.) are health sciences library workers familiar with the informed consent best practices and standards for the publication of medical images? and 2.) if so, how are they incorporating the recommendations into their professional work? Sub-questions were created based on different types of library duties the authors hypothesized would most likely incorporate the standards and best practices. The authors broadly defined the types of work as instruction, collections, and digital initiatives. The sub-questions were: 1.) Does knowledge of the standards and best practices influence instruction responsibilities? 2) Does knowledge of the standards and best practices influence collections responsibilities? 3) Does knowledge of the standards and best practices influence digital initiative responsibilities?

The goals of our research included understanding real-life phenomena and behavior and hopefully finding a way to improve lives. The paper will go on to describe the disseminated survey, the associated results, and the authors’ opinions on said results.

## METHODS

An exploratory quantitative study was conducted through a survey using Qualtrics software (Appendix A). Drafted by the authors, the survey investigated how aware our target population of health sciences library workers were of written, authoritative recommendations and best practices for obtaining informed consent to publish images of patients. Four non-health sciences librarians reviewed the initial drafted survey for face validity. Based on the testers’ feedback, revisions were made.

Following the revisions, the survey project was approved by the University of California, San Francisco IRB (registration #22-38211). Following IRB approval, the survey was disseminated across 20 North American listservs (see Appendix B) between April 4 and 7, 2023. A reminder was sent to the listservs between April 20 and 21. The survey closed on April 30, 2023. The authors were members of the chosen listservs or were able to connect with members who posted on their behalf. The listservs included the main Medical Library Association (MLA) listserv (MEDLIB-L), nine MLA chapter listservs, seven MLA caucus listservs, and three other listservs: University of California Libraries’ Health Sciences Selectors, the Association of College and Research Libraries (ACRL) Health Sciences Interest Group, and Electronic Resources in Libraries (ERIL).

Participation was voluntary, informed, and safe for the research subjects. The authors focused on disseminating the survey to health sciences library workers in the United States. The geographic limit was implemented because ethical standards and laws regarding medical image use and patient privacy vary widely across the world. If the respondent was outside of the United States, they were excluded. If a respondent was not a health sciences library worker, they were excluded and the survey ended. Incomplete responses were excluded. 91 respondents were included in the data for this paper, with 1 respondent removed from the dataset before analysis, as the respondent was not from the United States.

The survey consisted of 43 possible questions, 42 of which were multiple choice, and one was a free text response. Links to two informed consent guidelines were included at the start of the survey, COPE’s “Journals’ Best Practices for Ensuring Consent for Publishing Medical Case Reports” [25] and ICMJE’s “Protection of Research Participants” recommendation [24], so respondents could see examples of what was meant by informed consent standards and best practices for the publication of medical images. If the respondent said they were aware of these recommendations, the survey then asked how they incorporated that knowledge into instructional responsibilities, collections responsibilities, and/or digital initiative responsibilities, depending on what the respondent’s stated job duties were. If the respondent was not aware, the survey asked if they anticipated incorporating the recommendations and best practices into their job duties in the future now that they were aware. The survey also asked several demographic questions. Multiple choice questions had the option of a write-in response. The final survey question was a free text response box for respondents to add comments or questions.

All the statistics were evaluated using RStudio with R version 3.4.2 [41]. When possible, tables and charts were generated with relevant counts. Chi-squared tests (or Fisher’s Exact Test, the numerical alternative for chi-square, i.e., when any observed frequency was less than 5 in the table) were carried out to test if awareness of informed consent influenced the library worker’s job responsibilities. The significance level for all the tests was 0.05. The authors would like to note that though some results were statistically significant, it does not imply causation. The authors did not receive enough data in open-ended questions to warrant qualitative analysis.

## RESULTS

### Demographics

A total of 91 respondents were included in this data analysis. Among these respondents, 31% were research and instruction librarians. The distribution of various reported positions is shown in Table 1.

**Table 1:** The distribution of the different types of library positions.

Current Position	Percentages (Count)
Research and Instruction Librarian	31 (28)
Collection Development Librarian	1 (1)
Scholarly Communications Librarian	1 (1)
Liaison Librarian	12 (11)
Library Administrator	15 (14)
Clinical Librarian	1 (1)
Hospital Librarian	14 (15)
Information Specialist	6 (6)
Other	18 (18)

All respondents indicated that they had multiple responsibilities (instructional, collections, and/or digital initiatives) within their reported position. Over 25% of the respondents had worked for six to ten years in libraries and 22% of them worked for over twenty-five years.

When asked whether they were aware of any written, authoritative standards and best practices informed consent for the publication of medical images, 59% of the respondents said yes and 41% said no.

### Responsibilities vs. Awareness

#### Instructional Responsibilities

The authors then looked at the library workers who had instructional responsibilities (71 out of 91) and tested if awareness of the recommendations informed their instruction. The results of the Fisher’s Exact Test (p = [9.238e-09]) indicate a significant association between awareness and librarians involved in instruction. In other words, being a librarian involved in instruction indicates an awareness of the best practices described above. The authors also wanted to see, if they were not aware of the recommendations prior to the survey, whether the respondent would now have intentions to incorporate the recommendations in their job.

It was observed that over 50% of the library professionals with instructional responsibilities who were aware of the best practices and standards did not incorporate the recommendations in their responsibilities. The counts and percentages (shown in parenthesis) are provided in Table 2. The percentages were calculated using the row totals of 47 and 24 for each row.

**Table 2:** Frequency distribution of library workers with instructional responsibilities and their intent to incorporate recommendations.

	Incorporates best practices/ recommendations	Does not incorporate best practices/ recommendations	Unsure	Total
Aware before survey	22 (46.81%)	25 (53.19%)	0 (0%)	47
Not aware before survey	9 (37.50%)	2 (8.33%)	13 (54.17%)	24

#### Collections Responsibilities

Testing whether awareness informed collections duties (50 respondents out of 91), the authors again used the Fisher’s Exact Test for independence (*p* = [1.686e-05]) indicates a significant association between positions that involve collections responsibilities and awareness of informed consent. Table 3 shows that over 70% of the library professionals who were aware of recommendations before the survey do not incorporate said practices in their responsibilities. The percentages were calculated using row totals.

**Table 3:** Frequency distribution of library workers with collections responsibilities and their intent to incorporate recommendations.

	Incorporates best practices/ recommendations	Does not incorporate best practices/ recommendations	Unsure	Total
Aware before survey	9 (28%)	23 (72%)	0	32
Not aware before survey	4 (22%)	5 (28%)	9 (50%)	18

#### Digital Initiative Responsibilities

Repeating the Fisher’s Exact Test for independence (*p =* [0.09]) does not indicate a significant association between awareness and digital initiative responsibilities (13 respondents out of 91). Table 4 shows that over 60% of the library workers with digital initiative responsibilities who were aware of best practices before the survey incorporate them in their jobs. The percentages were calculated using row totals.

**Table 4:** Frequency distribution of library workers with digital initiative responsibilities and their intent to incorporate recommendations.

	Incorporates best practices/ recommendations	Does not incorporate best practices/ recommendations	Unsure	Total
Aware before survey	6 (67%)	3 (33%)	0 (0%)	9
Not aware before survey	2 (50%)	0 (0%)	2 (50%)	4

#### How Are Library Professionals Incorporating These Recommendations?

Respondents who had instructional responsibilities and were not aware of the standards (9.9%) indicated that they will incorporate the standards during one shot instruction sessions and by mentioning informed consent when advising others on research strategies, where to publish, and assessing journal quality. Respondents with digital initiative responsibilities who were aware of the standards (9.9%), reported that they will require informed consent from future contributors that meet these standards and best practices. Respondents with both collections and instructional responsibilities, who were not aware of the recommendations and did not wish to incorporate them (5.5%), indicated that they were not teaching the standards and best practices because they felt they were not relevant to their areas of responsibility. Unfortunately, the number of respondents who provided data regarding how they actually incorporate the recommendations in their library responsibilities was small (6.6%) and this result did not lend itself to further analysis.

The results of the survey in response to the primary research question were unexpected by the authors, as the majority of respondents were aware of the standards and best practices. As the original research question explored whether participants were aware or unaware of the standards, the authors are unable to explore why the standards are not currently being used and encourage future research on this important topic. Future research could greatly benefit the library profession by exploring how the recommendations are being incorporated by professionals in health sciences library settings.

Additionally, further research is also needed to better determine the deterrents preventing library workers from applying these recommendations to instruction, collections, and digital initiative work.

## DISCUSSION

The current incorporation of informed consent standards and best practices for the publication of medical images by health sciences library professionals appears to be limited. The data collected from the survey indicated that the majority of respondents (59%) were aware of the recommendations, but, with 41% of respondents not aware, there is significant room for further dissemination of the standards and best practices and for them to gain importance within librarianship. Since, in recent years, many libraries have committed to diversity, equity, and inclusion (DEI) initiatives and actions, including racial equity and social justice [42], the knowledge and application of informed consent standards and best practices for publishing medical images could be a key component of improvement within libraries. Specifically, within health sciences education, librarians providing instruction and consultation on these recommendations to researchers, faculty, and students who want to adhere to the best practices and standards and ensure their published materials are not endangering their patients could be a step towards ensuring the sustainability for DEI work within medical education [43]. This would have the added benefit of aligning librarians’ work within the larger framework of their institutions. Further, application of the recommendations could be developed into a possible standard to determine if DEI initiatives and actions are being met by libraries.

There were several limitations to this research, including a small sample pool, bias in the sample pool (i.e., lack of access to or awareness of other listservs could have caused sampling bias), and the general limitations associated with survey-based research. The authors strongly encourage future research on this topic, as there is a lack of richness in the data presented here. As is typical with survey research, a validated survey measurement would be helpful for future research as it would increase confidence in the quality of the data collected; however, there was no validated, appropriate measurement at the time that this research was conducted. In addition to suggesting the above first step of incorporating the recommendations into instruction and consultation, the authors believe that future studies could be extremely helpful for providing examples and improving the understanding of what library workers are currently doing successfully to incorporate informed consent recommendations for medical image publication into their professional work.

There are several options for further research on this important and timely topic, including where and how library professionals gained knowledge of the best practices and standards, as well as best practices for dissemination. The data presented here included 47% of respondents with instructional responsibilities who were aware of the best practices and standards, but did not incorporate them into their practice and, as this is exploratory research, this indicates this could be a fruitful area for future inquiry, particularly for qualitative research that explores why librarians do or do not incorporate informed consent recommendations into their instruction. The authors assumed instruction would be a key way for librarians to disseminate information regarding the recommendations, but dissemination through instruction was not widely reported among participants. Future research in this area could also focus on library professionals with roles other than instruction, collections, and digital initiatives to gain a better understanding of knowledge gaps. While this paper focused on health sciences library workers, future research could focus on general academic librarians who also have purchasing power for materials that include photographs of human subjects. The majority (68%) of respondents who had collections responsibilities also did not incorporate the best practices and standards into their professional practice, which was surprising to the authors, given the analytical and discerning nature of collection development work. Research on how to improve the incorporation of informed consent recommendations by librarians making collection development decisions could be another valuable study. Looking outside of libraries, further research could include collaborations with research offices and/or ethics programs to create wider, institutional-wide awareness of informed consent best practices and standards in both education and practice.

Libraries, as the primary purchasers of scholarly content, are uniquely positioned to support the informed consent standards and best practices for publishing medical images. Using this purchasing power, they could improve the adherence of these practices not only within their institutions, but also within publishing. If libraries collectively agreed that they would not subscribe to or purchase any publication that did not comply with informed consent recommendations, it is probable that significant changes would be made to ensure that publishers did not lose their library customers. In recent years, libraries and the institutions they serve have committed to various forms of DEI initiatives and aiding in the enforcement of informed consent recommendations would be a concrete way to prove those commitments while also enhancing patient safety. The authors urge library professionals to start conversations about how to best leverage the power and influence libraries possess to help bring about change that benefits historically marginalized populations, protects patients, and proves that institutional DEI goals are more than just vague promises.

## Data Availability

The survey results and code used to analyze the data are available via Dryad: https://datadryad.org/dataset/doi:10.5061/dryad.vmcvdnd17.

## References

[1] Ahmed S, Shipman A, Millington G, Langan EA, Ingram JR. Consent for publication: why it matters now more than ever. Br J Dermatol. 2020 Sep;183(3):409–10. 10.1111/bjd.19320 32880902

[2] Koch CA, Larrabee WF. Patient privacy, photographs, and publication. JAMA Facial Plast Surg. 2013;15(5):335–6. 10.1001/jamafacial.2013.1411 23887309

[3] Kunde L, McMeniman E, Parker M. Clinical photography in dermatology: ethical and medico-legal considerations in the age of digital and smartphone technology. Australas J Dermatol. 2013 Aug;54(3):192–7. 10.1111/ajd.12063 23713892

[4] Bhattacharya S. Clinical photography and our responsibilities. Indian J Plast Surg Off Publ Assoc Plast Surg India. 2014;47(3):277–80. 10.4103/0970-0358.146569 PMC4292101 25593409

[5] World Medical Association. WMA Declaration of Helsinki: Ethical Principles for Medical Research Involving Human Subjects [Internet]. 2013. Available from: https://www.wma.net/policies-post/wma-declaration-of-helsinki-ethical-principles-for-medical-research-involving-human-subjects/10.1001/jama.2013.281053 24141714

[6] Lakdawala N, Fontanella D, Grant-Kels JM. Ethical considerations in dermatologic photography. Clin Dermatol. 2012;30(5):486–91. 10.1016/j.clindermatol.2011.06.017 22902218

[7] Lau CK, Schumacher HHA, Irwin MS. Patients’ perception of medical photography. J Plast Reconstr Aesthetic Surg JPRAS. 2010 Jun;63(6):e507–511. 10.1016/j.bjps.2009.11.005 19959408

[8] Janagond A, Inamadar A. Clinical photography in dermatology: Perception and behavior of dermatologists – A pilot study. Indian Dermatol Online J. 2021;12(4):555. 10.4103/idoj.IDOJ_99_21 PMC8354416 34430459

[9] Roberts EA, Troiano C, Spiegel JH. Standardization of Guidelines for Patient Photograph Deidentification. Ann Plast Surg. 2016 Jun;76(6):611–4. 10.1097/SAP.0000000000000817 27015333

[10] Berle I. The principles of ethical practice in professional clinical photography. J Audiov Media Med. 2004 Mar;27(1):11–3. 10.1080/01405110310001658897 15203695

[11] Bennett KG, Bonawitz SC, Vercler CJ. Guidelines for the Ethical Publication of Facial Photographs and Review of the Literature. Cleft Palate-Craniofacial J Off Publ Am Cleft Palate-Craniofacial Assoc. 2019 Jan;56(1):7–14. 10.1177/1055665618774026 29715061

[12] Berle I. Clinical photography and patient rights: the need for orthopraxy. J Med Ethics. 2008 Feb;34(2):89–92. 10.1136/jme.2006.019166 18234945

[13] Kaliyadan F, Pasquali P, Ashique KT, Jayasree P, Al Dhafiri M. Clinical Images in Dermatology Manuscripts - A Study of Journal Submission Guidelines. Indian Dermatol Online J. 2022;13(6):734–6. 10.4103/idoj.idoj_223_22 PMC9650728 36386729

[14] Preston FG, Meng Y, Zheng Y, Hsuan J, Hamill KJ, McCormick AG. Informed Consent In Facial Photograph Publishing: A Cross-sectional Pilot Study To Determine The Effectiveness Of Deidentification Methods. J Empir Res Hum Res Ethics JERHRE. 2022 Jul;17(3):373–81. 10.1177/15562646221075459 PMC9136482 35068259

[15] Shinkai K, Bruckner AL, Robinson JK. Best Practices for Sharing Images in Clinical Care, Research, and Education—Protecting Patient Privacy. JAMA Dermatol. 2023 May;159(7):695–7. 10.1001/jamadermatol.2023.1080 37163261

[16] Creighton S, Alderson J, Brown S, Minto CL. Medical photography: ethics, consent and the intersex patient. BJU Int. 2002 Jan;89(1):67–71; discussion 71-72. 10.1046/j.1464-4096.2001.01809.x 11849163

[17] Nguyen TT, VanderWalde L, Bellavance E, Eisenhauer T, Hieken T, Johnson N, Kaufman DI, O’Neill J, Patten CR, Teller P, Tevis S, Sarantou T, Throckmorton A. Ethical Considerations of Medical Photography in the Management of Breast Disease. Ann Surg Oncol. 2018 Oct;25(10):2801–6. 10.1245/s10434-018-6603-6 29978370

[18] Palacios-González C. The ethics of clinical photography and social media. Med Health Care Philos. 2015 Feb;18(1):63–70. 10.1007/s11019-014-9580-y 25008805

[19] American Medical Association. Informed Consent. In: AMA Code of Medical Ethics [Internet]. [cited 2024 Feb 7]. Available from: https://code-medical-ethics.ama-assn.org/ethics-opinions/informed-consent

[20] Roguljić M, Peričić TP, Gelemanović A, Jukić A, Šimunović D, Buljan I, Marušić M, Marušić A, Wager E. What Patients, Students and Doctors Think About Permission to Publish Patient Photographs in Academic Journals: A Cross-Sectional Survey in Croatia. Sci Eng Ethics. 2020 Jun 1;26(3):1229–47. 10.1007/s11948-019-00134-y 31541413

[21] Office of the Federal Register NA and RA. 45 CFR § 164.522 - Rights to request privacy protection for protected health information. [Internet]. 2022 [cited 2024 Feb 25]. Available from: https://www.govinfo.gov/app/details/CFR-2022-title45-vol2/CFR-2022-title45-vol2-sec164-522/

[22] Taiwo T, Obiakor B, McClung S, Shinkai K. Informed Consent Practices for Publication of Patient Images in Dermatology Journals. JMIR Dermatology. 2025 Apr 18;8(1):e60795. 10.2196/60795 PMC12048787 40249653

[23] Roguljić M, Šimunović D, Poklepović Peričić T, Viđak M, Utrobičić A, Marušić M, Marušić A. Publishing Identifiable Patient Photographs in Scientific Journals: Scoping Review of Policies and Practices. J Med Internet Res. 2022 Aug 31;24(8):e37594. 10.2196/37594 PMC9475410 36044262

[24] International Committee of Medical Journal Editors. Protection of Research Participants [Internet]. Available from: https://www.icmje.org/recommendations/browse/roles-and-responsibilities/protection-of-research-participants.html

[25] Committee on Publication Ethics. Journals’ Best Practices for Ensuring Consent for Publishing Medical Case Reports [Internet]. 2016. Available from: https://publicationethics.org/files/Best_Practices_for_Ensuring_Consent_for_Publishing_Medical_Case_Reports_guidance_from_COPE.pdf

[26] Mavroforou A, Antoniou G, Giannoukas AD. Ethical and legal aspects on the use of images and photographs in medical teaching and publication. Int Angiol J Int Union Angiol. 2010 Aug;29(4):376–9. 20671657

[27] Roguljić M, Buljan I, Veček N, Dragun R, Marušić M, Wager E, Marušić A. Deidentification of facial photographs: a survey of editorial policies and practices. J Med Ethics. 2022 Jan;48(1):56–60. 10.1136/medethics-2019-105823 32253363

[28] Berle I. The ethical context of clinical photography. J Audiov Media Med. 2002 Jan 1;25(3):106–9. 10.1080/014051102320376816 12626059

[29] Kim W, Sivesind T. Patient Perceptions of Dermatologic Photography: Scoping Review. JMIR Dermatol. 2022 Jan 26;5(1):e33361. 10.2196/33361 PMC10334897 37632867

[30] Marshall Z, Brunger F, Welch V, Asghari S, Kaposy C. Open Availability of Patient Medical Photographs in Google Images Search Results: Cross-Sectional Study of Transgender Research. J Med Internet Res. 2018 Feb 26;20(2):e70. 10.2196/jmir.8787 PMC5847816 29483069

[31] Leger MC, Wu T, Haimovic A, Kaplan R, Sanchez M, Cohen D, Leger EA, Stein JA. Patient Perspectives on Medical Photography in Dermatology. Dermatol Surg. 2014 Sep;40(9):1028. 10.1097/01.DSS.0000452632.22081.79 25099296

[32] Devakumar D, Brotherton H, Halbert J, Clarke A, Prost A, Hall J. Taking ethical photos of children for medical and research purposes in low-resource settings: an exploratory qualitative study. BMC Med Ethics. 2013 Jul 9;14:27. 10.1186/1472-6939-14-27 PMC3750443 23835013

[33] Burns K, Belton S. “Click first, care second” photography. Med J Aust. 2012 Sep 3;197(5):265. 10.5694/mja12.10638 22938110

[34] Burns K, Belton S. Clinicians and their cameras: policy, ethics and practice in an Australian tertiary hospital. Aust Health Rev. 2013 Jun 19;37(4):437–41. 10.1071/AH12039 23777890

[35] Petersilge CA. Fundamentals of Enterprise Photodocumentation: Connecting the Clinical and Technical—a Review of Key Concepts. J Digit Imaging. 2019 Dec 1;32(6):1052–61. 10.1007/s10278-019-00212-4 PMC6841903 31011957

[36] Kovarik CL, Sanabria B, Stoff BK. Image Consent and the Development of Image-Based Artificial Intelligence. JAMA Dermatol. 2022 May 1;158(5):589. 10.1001/jamadermatol.2022.0689 35416918

[37] Taylor D, Foster E, Dunkin CSJ, Fitzgerald AM. A study of the personal use of digital photography within plastic surgery. J Plast Reconstr Aesthet Surg. 2008 Jan 1;61(1):37–40. 10.1016/j.bjps.2007.02.033 17766207

[38] Chan N, Charette J, Dumestre DO, Fraulin FO. Should “smart phones” be used for patient photography? Plast Surg Oakv Ont. 2016;24(1):32–4. PMC4806754 27054136

[39] Bhangoo P, Maconochie IK, Batrick N, Henry E. Clinicians taking pictures—a survey of current practice in emergency departments and proposed recommendations of best practice. Emerg Med J. 2005 Nov 1;22(11):761–5. 10.1136/emj.2004.016972 PMC1726600 16244330

[40] Teven CM, Park JE, Song DH. Social Media and Consent: Are Patients Adequately Informed? Plast Reconstr Surg. 2017 Nov;140(5):770e. 10.1097/PRS.0000000000003805 28753143

[41] R: The R Project for Statistical Computing [Internet]. [cited 2024 Mar 6]. Available from: https://www.r-project.org/

[42] Bresnahan M. Library Diversity and Inclusion Statements in Action. J Libr Adm. 2022 May 19;62(4):419–37.

[43] Yepes-Rios M, Lad S, Dore S, Thapliyal M, Baffoe-Bonnie H, Isaacson JH. Diversity, equity, and inclusion: one model to move from commitment to action in medical education. SN Soc Sci. 2023 Mar 11;3(3):61. 10.1007/s43545-023-00650-6 PMC10005912 36937456

