# Supplementary material for: Health sciences librarians’ awareness and incorporation of informed consent standards for medical image publication: a preliminary study
Source: J Med Libr Assoc. 2026 Oct 14;114(4):401–9. doi: 10.5195/jmla.2026.1949 (PMC13670234; doi:10.5195/jmla.2026.1949)
Supplement: Supplementary file 1 [file jmla-114-4-401-s01.pdf]

Appendix A - Survey

Hello,

We are doing a research study about informed consent standards for the publication of medical images. We would like you to participate in this study as a health sciences library worker.

If you choose to be in the study, you will complete an online survey. It will take about 10 minutes to complete.

You can skip questions that you do not want to answer or stop the survey at any time. The survey is anonymous, and no one will be able to link your answers back to you. Please do not include your name or other information that could be used to identify you in the survey responses.

Please contact me with questions about this study. If you have questions or concerns about your rights as a research participant, you can call the UCSF Institutional Review Board at 415-476-1814. Being in this study is optional. If you want to participate, please indicate your consent on the next screen.

Thank you,
Sarah McClung, MIS
Head of Collection Development
University of California, San Francisco Library
sarah.mcclung@ucsf.edu

I have read the informed consent for this study and I agree to the informed consent.

- Yes
- No

Do you currently work in a library that serves students, faculty, clinicians, practitioners, or researchers in the health or medical sciences?

- Yes
- No

Are you aware of any written, authoritative recommendations and best practices for obtaining informed consent to publish images of a research subject? Examples include "Journals' Best Practices for Ensuring Consent for Publishing Medical Case Reports" by the Committee on Publication Ethics (COPE) and "Protection of Research Participants Recommendation" by the International Committee of Medical Journal Editors (ICMJE). It is recommended that survey respondents at least skim these short documents so that they can more accurately answer the remaining questions.

- Yes
- No

Are instruction responsibilities part of your current role?

- Yes
- No

Now that you are aware of these recommendations and best practices, do you anticipate incorporating them into your library instruction, reference, and/or consultations in the future?

- Yes

- No
- Unsure

Where and/or how do you anticipate incorporating these recommendations and best practices into your library instruction, reference, and/or consultations in the future? Select all that apply.

- One shot instruction sessions
- Semester long courses
- Courses in a learning management system (LMS), such as Canvas or Blackboard
- Live Demos
- Include in list(s) of resources, such a handouts or LibGuides
- Digital Learning objects, such as videos or online tutorials
- Mention informed consent and images when advising others on research strategies
- Mention informed consent and images when advising others on where to publish
- Mention informed consent and images when advising others on assessing journal quality
- Unsure
- Other

Why do you not anticipate incorporating these recommendations and best practices into your library instruction, reference, and/or consultations in the future? Select all that apply.

- Not relevant to my instruction area(s)
- I don't believe this is a pressing issue
- I don't know how
- I don't feel comfortable with the concepts
- I want to develop a better understanding of this topic before I include this into my professional practice
- Institutional support if unknown or doubtful
- Other

Are collections responsibilities part of your current role?

- Yes
- No

Now that you are aware of these recommendations and best practices, do you anticipate that this knowledge will affect your collections work in the future?

- Yes
- No
- Unsure

How do you anticipate incorporating these recommendations and best practices into your collections work in the future? Select all that apply

- Look for evidence of informed consent requirements from publishers/associations that meet these recommendations and best practices for future purchases or subscriptions
- Look for evidence of informed consent requirements from publishers/associations that meet these recommendations and best practices for current purchases and subscriptions
- Unsubscribe from resources that do not meet the recommendations and best practices
- Do not purchase resources that do not meet the recommendations and best practices
- Incorporate these recommendations and best practices into your collection development and/or purchasing policies
- Mention informed consent when speaking about and recommending library resources to stakeholders
- Contact publishers/associations and encourage them to adopt these recommendations and best practices if they haven't already
- Unsure

- Other

Why do you not anticipate incorporating these recommendations and best practices into your collections work in the future? Select all that apply.

- Not relevant to my collection area(s)
- I don’t believe this is a pressing issue
- I don’t know how
- I don’t feel comfortable enough with the concepts
- I want to develop a better understanding of this topic before I include this into my professional practice
- Institutional support is unknown or doubtful
- Other

Are digital initiative responsibilities part of your current role? Examples include institutional repositories (IRs), in-house image banks, and archival scanning.

- Yes
- No

Now that you are aware of these recommendations and best practices, do you anticipate that this knowledge will affect your digital initiatives work in the future?

- Yes
- No
- Unsure

How do you anticipate incorporating these recommendations and best practices into your digital initiatives work in the future? Select all that apply.

- Require informed consent from future contributors that meet these recommendations and best practices
- Look for evidence of informed consent from current contributors that meet these recommendations and best practices
- Remove materials that do not meet the recommendations and best practices
- Do not include future materials that do not meet the recommendations and best practices
- Incorporate these recommendations and best practices into relevant policies
- Mention informed consent when speaking with stakeholders
- Contact current and future contributors and encourage them to adopt these recommendations and best practices if they haven’t already
- Unsure
- Other

Why do you not anticipate incorporating these recommendations and best practices into your digital initiatives work in the future? Select all that apply.

- Not relevant to my digital initiatives area(s)
- I don’t believe this is a pressing issue
- I don’t know how
- I don’t feel comfortable enough with the concepts
- I want to develop a better understanding of this topic before I include this into my professional practice
- Institutional support is unknown or doubtful
- Other

Now that you are aware of these recommendations and best practices, do you anticipate this knowledge will affect your job duties in ways not previously addressed in this survey? Why or why not?

Are instruction responsibilities part of your current role?
- o Yes
- o No

Do you incorporate these recommendations and best practices into your library classes, reference, and/or consultations?
- o Yes
- o No

Why do you not incorporate these recommendations and best practices into your library instruction, reference, and/or consultations? Select all that apply.
- ▪ Not relevant to my instruction area(s)
- ▪ I don't believe this is a pressing issue
- ▪ I don't know how
- ▪ I don't feel comfortable enough with the concepts
- ▪ I want to develop a better understanding of this topic before I include this into my professional practice
- ▪ Institutional support is unknown or doubtful
- ▪ Other

How do you incorporate these recommendations and best practices into your library classes and/or consultations? Select all that apply.
- ▪ One shot instruction sessions
- ▪ Semester long courses
- ▪ Courses in a learning management system (LMS), such as Canvas or Blackboard
- ▪ Live demos
- ▪ Include in list(s) of resources, such as handouts or LibGuides
- ▪ Digital learning objects, such as videos or online tutorials
- ▪ Mention informed consent when advising others on research strategies
- ▪ Mention informed consent when advising others on where to publish
- ▪ Mention informed consent when advising others on assessing journal quality
- ▪ Other

Are collections responsibilities part of your current role?
- o Yes
- o No

Does knowledge of these recommendations and best practices affect your collections work?
- o Yes
- o No

Why do you not incorporate these recommendations and best practices into your collections work? Select all that apply.
- ▪ Not relevant to my collections area(s)
- ▪ I don't believe this is a pressing issue
- ▪ I don't know how
- ▪ I don't feel comfortable enough with the concepts
- ▪ I want to develop a better understanding of this topic before I include this into my professional practice
- ▪ Institutional support is unknown or doubtful
- ▪ Other

How do you incorporate these recommendations and best practices into your collections work? Select all that apply.

- Look for evidence of informed consent requirements from publishers/associations that meet these recommendations and best practices for future purchases or subscriptions
- Look for evidence of informed consent requirements from publishers/associations that meet these recommendations and best practices for current purchases and subscriptions
- Unsubscribe from resources that do not meet the recommendations and best practices
- Do not purchase resources that do not meet the recommendations and best practices
- Incorporate these recommendations and best practices into your collection development and/or purchasing policies
- Mention informed consent when speaking about and recommending library resources to stakeholders
- Contact publishers/associations and encourage them to adopt these recommendations and best practices if they haven't already
- Unsure
- Other

Are digital initiative responsibilities part of your current role? Examples include institutional repositories (IRs), in-house image banks, and archival scanning.

- Yes
- No

Does knowledge of these recommendations and best practices affect your digital initiatives work?

- Yes
- No

Why do you not incorporate these recommendations and best practices into your digital initiatives work? Select all that apply.

- Not relevant to my digital initiatives area(s)
- I don't believe this is a pressing issue
- I don't know how
- I don't feel comfortable enough with the concepts
- I want to develop a better understanding of this topic before I include this into my professional practice
- Institutional support is unknown or doubtful
- Other

How do you incorporate these recommendations and best practices into your digital initiatives work? Select all that apply.

- Require informed consent from future contributors that meet these recommendations and best practices
- Look for evidence of informed consent from current contributors that meet these recommendations and best practices
- Remove materials that do not meet the recommendations and best practices
- Do not include future materials that do not meet the recommendations and best practices
- Incorporate these recommendations and best practices into relevant policies
- Mention informed consent when speaking with stakeholders
- Contact current and future contributors and encourage them to adopt these recommendations and best practices if they haven't already
- Other

Does knowledge of these recommendations and best practices affect your job duties in ways not previously addressed in this survey? Why or why not?

Do you identify as transgender?
- Yes
- No
- Prefer not to disclose

What is your gender identity? Select all that apply.
- Agender
- Genderqueer or genderfluid
- Māhū
- Man
- Muxe
- Non-binary
- Questioning or unsure
- Two-spirit
- Woman
- Prefer not to disclose
- Additional gender category/identity not listed. Please specify:

What is your race? Select all that apply; these categories are based on the Federal Government official standards.
- American Indian or Alaska Native. A person having origins in any of the original peoples of North and South America (including Central America), and who maintains tribal affiliation or community attachment.
- Asian. A person having origins in any of the original peoples of the Far East, Southeast Asia, or the Indian subcontinent including, for example, Cambodia, China, India, Japan, Korea, Malaysia, Pakistan, the Philippine Islands, Thailand, and Vietnam.
- Black or African American. A person having origins in any of the Black racial groups of Africa. Terms such as "Haitian" or "Afro-Caribbean" can be used in addition to "Black or African American."
- Hispanic or Latino. A person of Cuban, Mexican, Puerto Rican, South or Central American, or other Spanish culture or origin, regardless of race.
- Native Hawaiian or Pacific Islander. A person having origins in any of the original peoples of Hawaii, Guam, Samoa, or other Pacific Islands.
- White. A person having origins in any of the original peoples of Europe, the Middle East, or North Africa.
- Some other race

In which country do you currently reside?
Afghanistan ....Zimbabwe

How long have you worked in libraries?
- 0-5 years
- 6-10 years
- 11-15 years
- 15-20 years
- 21-25 years
- Over 25 years

How long have you worked in your current position?
- 0-5 years
- 6-10 years
- 11-15 years

- 16-20 years
- 21-25 years
- Over 25 years

What is your age?
- 18-24
- 25-30
- 31-35
- 36-40
- 41-45
- 46-50
- 51-54
- 55-60
- Over 60

What is your current position?
- Research and Instruction Librarian
- Collection Development Librarian
- Acquisitions Librarian
- Scholarly Communications Librarian
- Liaison Librarian
- Library Administrator
- Clinical Librarian
- Hospital Librarian
- Institutional Repository Librarian
- Archivist
- Information Specialist
- Other

At what type of library do you currently work?
- Academic, serving predominantly undergraduate programs
- Academic, serving predominantly graduate programs
- Academic, serving predominantly health sciences programs
- Hospital
- Corporate
- Other

Please share any questions or comments you may have about the topic of recommendations and best practices for informed consent of published medical images.
