# Supplementary material for: Health sciences librarians’ awareness and incorporation of informed consent standards for medical image publication: a preliminary study
Source: J Med Libr Assoc. 2026 Oct 14;114(4):401–9. doi: 10.5195/jmla.2026.1949 (PMC13670234; doi:10.5195/jmla.2026.1949)
Supplement: Supplementary file 2 [file jmla-114-4-401-s02.pdf]

Appendix B - Survey Distribution

The survey was distributed to 20 listservs

1	MEDLIB-L: An Email Discussion LIst for Medical Librarians	https://www.mlanet.org/p/cm/ld/fid=377
MLA Chapters (9 of 12 chapters)		
2	Northern California and Nevada Medical Library Group (NCNMLG)	https://ncnmlg.mlanet.org/main/
3	Medical Library Group of Southern California and Arizona (MLGSCA)	https://mlgsca.mlanet.org/beta/
4	Pacific Northwest Chapter of MLA (PNC)	https://pncmla.org/
5	Southern Chapter (SC)	https://southernchaptermla.wildapricot.org/
6	Liberty Chapter (merged NY-NJ and Philadelphia Regional Chapters)	https://libertymla.org/page-1471652
7	South Central Chapter (SCC)	https://www.sccmla.org/
8	Midcontinental Chapter (MCMLA)	https://macmla.org/
9	Midwest Chapter	https://mcmla45.wildapricot.org/
10	North Atlantic Health Sciences Libraries (NAHSL)	https://nahsl.libguides.com/NAHSL
MLA Caucuses		
11	Collection Development	https://www.mlanet.org/p/cm/ld/fid=391
12	Research	https://www.mlanet.org/p/cm/ld/fid=503
13	Libraries in Health Sciences Curriculums	https://www.mlanet.org/page/caucus-libraries-in-hscurriculums
14	Medical Library Education	https://www.mlanet.org/p/cm/ld/fid=533
15	Osteopathic Libraries	https://www.mlanet.org/page/caucus-ol
16	Technology in Education	https://www.mlanet.org/page/emts

17	User Experience	https://www.mlanet.org/page/user-experience-caucus
Other Listservs		
18	UC Libraries Health & Life Sciences (UCHLS) Common Knowledge Group (CKG)	https://libraries.universityofcalifornia.edu/ckg/
19	The Association of College and Research Libraries (ACRL) Health Sciences Interest Group	https://connect.ala.org/acrl/communities/community-home?CommunityKey=8fdcf5eb-1258-4a06-ae0d-c484ac02cd16
20	Electronic Resources in Libraries (ERIL)	http://lists.eril-l.org/listinfo.cgi/eril-l-eril-l.org
